# Mitochondrial DNA Supplementation of Oocytes Has Downstream Effects on the Transcriptional Profiles of *Sus scrofa* Adult Tissues with High mtDNA Copy Number

**DOI:** 10.3390/ijms24087545

**Published:** 2023-04-19

**Authors:** Takashi Okada, Alexander Penn, Justin C. St. John

**Affiliations:** Mitochondrial Genetics Group, School of Biomedicine, Faculty of Health and Medical Sciences, The University of Adelaide, Adelaide, SA 5000, Australia

**Keywords:** autologous, heterologous, glyoxylate metabolism, immune response, imprinted gene, mitochondrial DNA, mitochondrial DNA supplementation, oxidative phosphorylation, *Sus scrofa*, transcriptome analysis

## Abstract

Oocytes can be supplemented with extra copies of mitochondrial DNA (mtDNA) to enhance developmental outcome. Pigs generated through supplementation with mtDNA derived from either sister (autologous) or third-party (heterologous) oocytes have been shown to exhibit only minor differences in growth, physiological and biochemical assessments, and health and well-being do not appear affected. However, it remains to be determined whether changes in gene expression identified during preimplantation development persisted and affected the gene expression of adult tissues indicative of high mtDNA copy number. It is also unknown if autologous and heterologous mtDNA supplementation resulted in different patterns of gene expression. Our transcriptome analyses revealed that genes involved in immune response and glyoxylate metabolism were commonly affected in brain, heart and liver tissues by mtDNA supplementation. The source of mtDNA influenced the expression of genes associated with oxidative phosphorylation (OXPHOS), suggesting a link between the use of third-party mtDNA and OXPHOS. We observed a significant difference in parental allele-specific imprinted gene expression in mtDNA-supplemented-derived pigs, with shifts to biallelic expression with no effect on expression levels. Overall, mtDNA supplementation influences the expression of genes in important biological processes in adult tissues. Consequently, it is important to determine the effect of these changes on animal development and health.

## 1. Introduction

Amongst other processes, one of the key roles of mitochondria is energy production through respiration and oxidative phosphorylation (OXPHOS) [1]. Most of the mitochondrial proteins are encoded by the nuclear genome, including those associated with the electron transfer chain (>1000 genes). However, mitochondrial DNA (mtDNA) encodes 13 of the protein-coding genes of the electron transport chain, and 22 tRNAs and 2 rRNAs [2,3,4], and mutation and deletion of this genome can lead to debilitating and severe phenotypes [5,6]. Given that the electron transfer chain is encoded by two genomes, crosstalk between the nuclear and mitochondrial genomes is essential for mitochondrial function so that cells can function effectively and respond to environmental and developmental stimuli [7,8]. This cooperation is established during very early development when the two genomes first interact and is maintained throughout development and in adulthood [9].

Cellular response and function require appropriate levels of OXPHOS-derived ATP, and mtDNA copy number per cell varies amongst cell and tissue types dependent on their specialised functions [10,11]. For example, brain, heart and liver have higher mtDNA copy number per cell (>3000) because of their high energy requirements to mediate complex functions such as action potentials in some cells of the heart and brain. Mammalian oocytes contain the highest levels of mtDNA (>150,000). This population of mtDNA is considered to be an investment in subsequent developmental events [12] as there are no major mtDNA replication events in cells giving rise to the embryo proper until post-gastrulation [13,14]. As a result, mtDNA copy number decreases in each newly formed embryonic cell by half due to cell division [15,16]. Consequently, oocytes with low levels of mtDNA copy number, a form of mitochondrial deficiency, frequently fail to fertilise or arrest during preimplantation development and this is now considered to be one of the causes of female factor infertility [13,14,17,18,19].

Ooplasmic transfer and mitochondrial supplementation of oocytes have been shown to improve rates of fertilisation and development of mammalian embryos [15,20,21,22,23,24]. Furthermore, supplementation in combination with intracytoplasmic sperm injection (ICSI) of mtDNA-deficient pig oocytes triggers a mtDNA replication event by the two-cell stage of embryo development that increases mtDNA content by 4.4-fold and improves embryo quality and developmental rates [20]. However, there was little difference in the number of mitochondria between the ICSI-derived two-cell stage embryos generated from mtDNA-deficient and mtDNA-normal oocytes [20]. Since a major activation of abundant transcription occurs at a later stage of embryo development, namely the four- to eight cell stage [25,26], it is unlikely that there would be sufficient transcripts and proteins to generate large numbers of mitochondria in two-cell stage embryos. Furthermore, mtDNA supplementation modulates the methylation status of *DNA polymerase gamma* (*POLG*), which is one of the key factors involved in mtDNA replication, with significant correlations between DNA methylation of *POLG* and mtDNA copy number in developing embryonic cells [27]. We have also shown in a pig model that, at the blastocyst stage, supplementation alters the DNA methylation profiles of over 2000 nuclear genomic regions which, in turn, influenced the expression of a subset of nuclear-encoded genes [20,27,28]. In this respect, the expression of genes associated with haematological system development, immune cell trafficking and inflammatory response pathways were most affected. Therefore, it is evident that mtDNA supplementation influences the DNA methylation and gene expression profiles of developing embryos, indicating that the process mediates genomic rather than metabolic events. Consequently, we refer to the supplementation process as mtDNA supplementation although the mitochondrial genome is packaged in purified populations of mitochondria. This preserves the integrity of the mitochondrial genome and protects it from the cytosolic enzymes of the oocyte that would otherwise likely result in its destruction.

More recently, we have reported on the generation of pigs produced through mtDNA supplementation in combination with ICSI where the mtDNA was derived from either sister (autologous) or third-party (heterologous) oocytes [29]. Despite changes in epigenetic marks and gene expression at the blastocyst stage of development (the final stage of preimplantation development) [20,27,28], pigs derived from mtDNA supplementation revealed only minor differences in growth, and their health and well-being did not seem to be affected [29]. Both in vitro fertilisation and ICSI have been reported to affect imprinting, DNA methylation and gene expression compared to the children born from natural conception [30,31,32,33]. We have shown in pigs derived from mtDNA supplementation that imprint control regions of imprinted genes showed no difference in DNA methylation patterns from naturally conceived control pigs [29]. However, it is still largely unknown if: (1) altered gene expression identified during early development following mtDNA supplementation persists and affects gene expression in adult tissues; (2) supplementation through third-party (heterologous) and autologous mtDNA differently affect gene expression; and (3) imprinted genes are expressed differently in adult tissues.

In this study, we addressed these questions by transcriptome analyses of adult pig tissues derived from mtDNA supplementation compared to naturally conceived control pigs. We investigated brain, heart, and liver tissues as they contain high numbers of mtDNA copy per cell [10,11] and are reliant on OXPHOS for ATP production. Differentially expressed genes between control and mtDNA-supplemented-derived pig tissues were identified and analysed for association with specific biological functions. A similar comparison was conducted between autologous and heterologous mtDNA-supplemented-derived pig tissues. We also investigated levels of imprinted gene expression and parental allele-specific expression patterns by analysing single nucleotide polymorphism in the transcripts. Our analysis uncovered genes and functional pathways affected in the adult tissues of pigs arising from mtDNA supplementation and between autologous- and heterologous-derived tissues.

## 2. Results

### 2.1. Transcriptome Data from mtDNA-Supplemented Adult Pig Tissues with High mtDNA Copy Number

**Figure 1:**
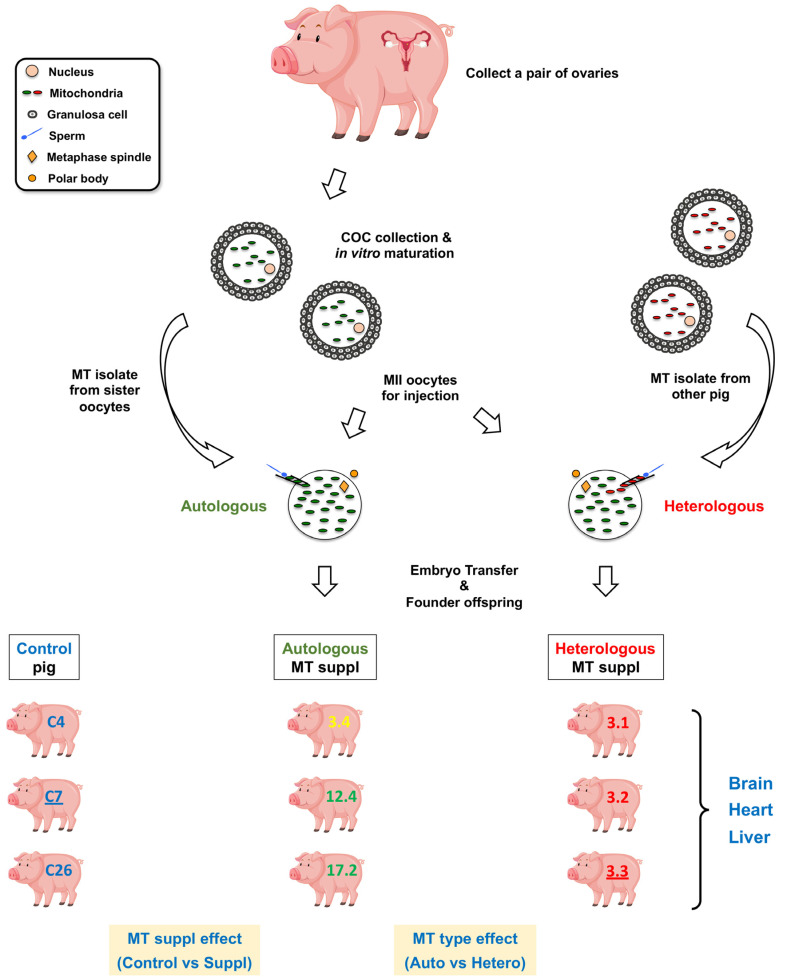
Schematic representation of the production of autologous and heterologous mtDNA-supplemented-derived offspring, RNAseq sample collection and data analysis strategy. For autologous mtDNA supplementation, ‘sister’ oocytes from the same ovary pair were used for both preparation of the mitochondrial (MT) isolate and the supplementation process. For heterologous supplementation, the MT isolate was derived from third-party pig oocytes. Three sets of mtDNA-supplemented founder offspring were generated [29] as illustrated. Underlined pig IDs represent female offspring. Heart, brain and liver tissues were collected from control and mtDNA-supplemented-derived pigs for RNAseq. The effect of mtDNA supplementation was investigated by comparing the three control (pig IDs: C4, C7 and C26) and six mtDNA-supplemented (autologous—3.4, 12.4 and 17.2; and heterologous—3.1, 3.2 and 3.3) pigs (Table S1). The effect of the source of mtDNA supplementation was analysed by comparing tissues from autologous and heterologous pigs. Pig and ovary images were designed by brgfx/Freepik.

We collected brain, heart and liver tissues from adult pigs for transcriptome analysis as illustrated in Figure 1. In all, we collected tissue samples from three heterologous (Pigs 3.1, 3.2 and 3.3) and three autologous mtDNA-supplemented-derived pigs (pigs 12.4, 17.2 and 3.4) and three control pigs (C4, 7 and 26). Pig 3.4 was initially thought to be heterologous (Figure 1), however, mtDNA genotyping indicated that the mtDNA supplement originated from oocytes possessing identical mtDNA genotypes to the donor oocyte [29]. Therefore, we included pig 3.4 as an autologous mtDNA-supplemented-derived pig for transcriptome comparisons.

**Figure 2:**
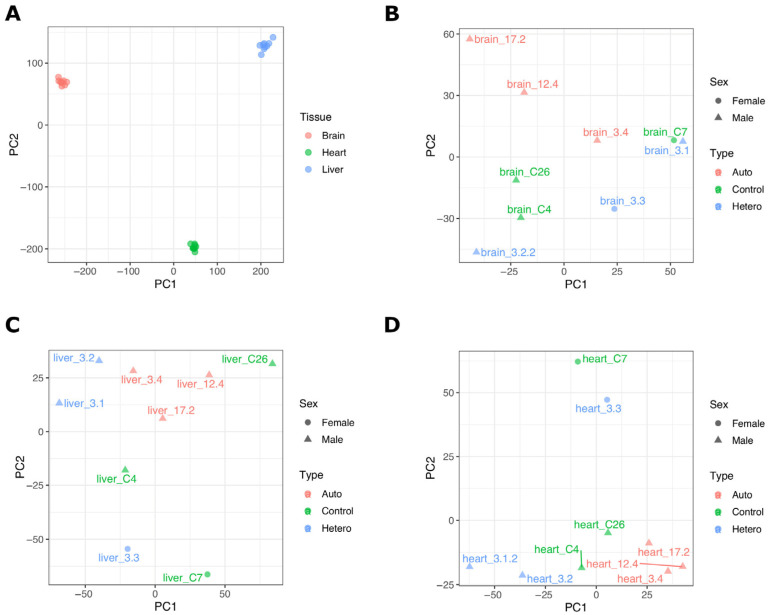
Principal component analysis (PCA) of RNAseq data for liver, heart and brain tissues. (**A**) PCA plot for all tissues used in this study. (**B**) Brain tissue from control and mtDNA-supplemented-derived pigs. (**C**) Liver tissue from control and mtDNA-supplemented-derived pigs. (**D**) Heart tissue from control and mtDNA-supplemented-derived pigs. In (**B**–**D**), control (green), and autologous (orange) and heterologous (blue) mtDNA-supplemented-derived pig data are indicated by different colours, and the gender of each pig is shown by a triangle (male) or circle (female). Sample metadata and summary statistics for RNAseq data are shown in Table S1.

Next-generation sequences of RNA samples resulted in 49–111M paired-end reads per sample after adapter trimming (Table S1). Generally, more than 85% of the sequence reads were successfully mapped to the *Sus scrofa* reference genome, and 21–46M reads per sample had functional annotations, which were therefore used for analysis of differentially expressed genes (DEGs). Principal component analysis (PCA) showed distinct RNAseq profiles for each tissue type (Figure 2A) with high correlations between individual samples of the same tissues (*R* > 0.95). We have previously analysed heart RNAseq data from these pigs but only in the context of mtDNA supplementation and controls [29]. Here, we have not only compared the additional mtDNA-supplemented tissues to controls but have also compared heterologous and autologous samples to determine whether the source of mtDNA for supplementation would impact on tissue-specific gene expression (Figure 1). Out of nine tissue RNAseq data sets, two were derived from female pigs (C7 and 3.3) and formed a distinctive group as shown in the PCA plots, i.e., separated from the male pigs, especially for the heart and liver data sets (Figure 2C,D). Therefore, sex was included as a factor in the linear model for data correction, when appropriate.

### 2.2. mtDNA Supplementation Affects mtDNA-Encoded Transcript Levels in Adult Tissues with High mtDNA Copy Number

In order to determine whether the addition of extra copies of mtDNA impacted on each tissue’s overall gene expression profiles, we investigated whether supplemented pigs differentially expressed any genes relative to controls. In brain, 19 genes were differentially expressed between control and mtDNA-supplemented-derived (autologous and heterologous) pigs (Table 1, Figures S1A and S2A). These included three mtDNA encoded transcripts (2× tRNA and 1× rRNA), nine nuclear protein coding and six long non-coding RNAs (lncRNA). In liver, 19 DEGs were identified including one mtDNA encoded tRNA, 13 nuclear protein coding and three lncRNAs (Table 1, Figures S1C and S2C). In heart tissue, we identified 18 DEGs including four mtDNA encoded transcripts (3× tRNA and ATP8), 12 nuclear protein coding and two lncRNAs (Table 1, Figures S1B and S2B). Five of these transcripts were previously identified as DEGs in heart [28]; however, the remainder are novel due to the use of a different annotation database and two additional data sets. It is interesting to note that pseudogene transcript ENSSSCG00000002529 was significantly downregulated in all three mtDNA-supplemented-derived tissues (Table 1 and Figure S1). Overall, eight out of the 56 DEGs identified in the tissues of pigs generated by mtDNA supplementation were mtDNA encoded. To this extent, we have observed a similar trend in mtDNA-supplemented-derived blastocysts [28], suggesting that the effect of mtDNA supplementation on levels of mtDNA- and nuclear-encoded transcripts persists in adult tissues.

### 2.3. mtDNA Supplementation Influences Glyoxylate Metabolism and Interferon Signalling Pathways

**Figure 3:**
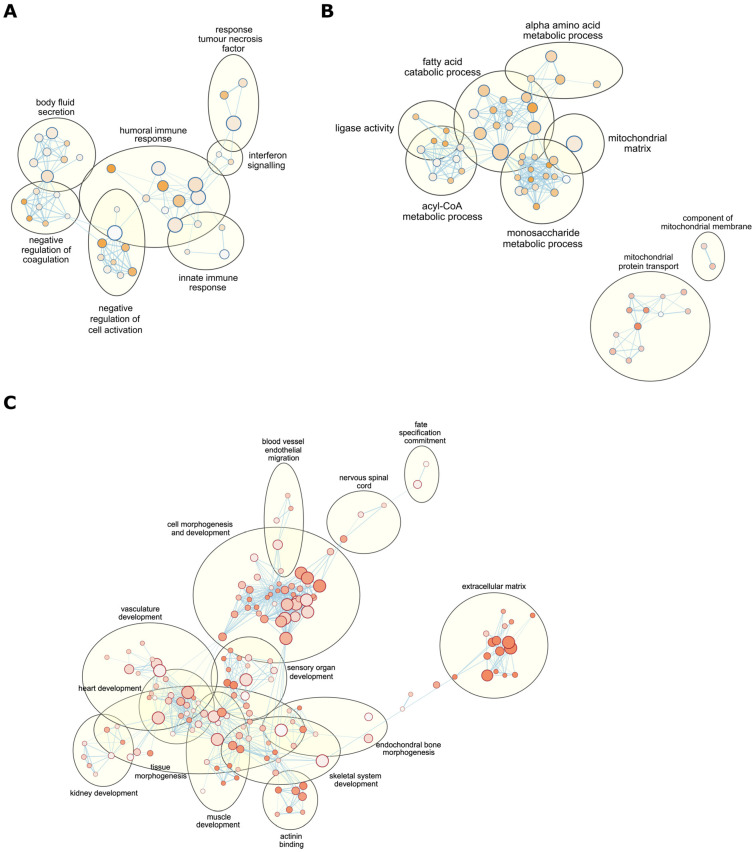
Functional pathway enrichment analysis displaying gene ontology (GO) biological process term networks. (**A**) The largest network associated with immune response, interferon signalling, and coagulation found in differentially expressed genes (DEGs) identified in brain tissue from mtDNA-supplemented and control pigs; (**B**) the largest network associated with fatty acid, acyl-CoA, monosaccharide metabolic processes (top) and mitochondrial protein transport related network (bottom) identified from heart DEGs between mtDNA-supplemented and control tissues; and (**C**) the largest network associated with morphogenesis and development for tissues and organs found in liver DEGs between mtDNA-supplemented and control tissues. GO terms with related function were grouped by Cytoscape auto-annotation function, and group annotation titles were given automatically or manually. Each circle represents an individual GO term, and the colour and size of a circle indicate the level of significance by pathway enrichment analysis and the number of genes associated with the GO term, respectively.

Since differential gene expression associated with mtDNA supplementation could influence specific functions in adult tissues, we investigated this by undertaking enrichment analysis using gene ontology (GO) biological process terms [34] using whole DEG data sets (see Methods). GO terms associated with immune response, interferon signalling and coagulation formed the largest functional network affected by mtDNA supplementation in brain tissue (Figure 3A). Fatty acid, acyl-CoA, monosaccharide metabolic processes and mitochondrial protein transport were largely affected in heart (Figure 3B). The liver showed that genes associated with morphogenesis and development for various tissues and organs were affected by mtDNA supplementation (Figure 3C). Consequently, the impact of mtDNA supplementation at a functional level varied amongst different tissues.

**Figure 4:**
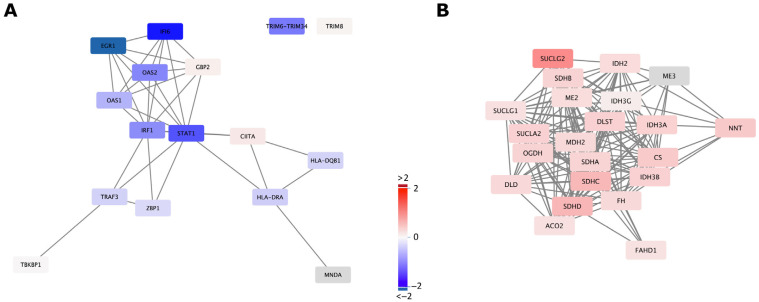
Gene network and differential gene expression of functional pathways identified by enrichment analysis. (**A**) Interferon signalling pathway (R-HSA-913531) genes expressed in tissues were used to construct a protein interaction network by STRING. Levels of differential gene expression between control and mtDNA-supplemented-derived liver are shown as fold change relative to control by colour scale (right). (**B**) The tricarboxylic acid (TCA) cycle pathway (R-HSA-71403) genes expressed in tissues were used to construct a protein interaction network. Levels of differential gene expression between heterologous and autologous mtDNA-supplemented-derived brain are shown as fold change relative to autologous mtDNA-supplemented-derived brain by colour scale. The same colour scale was used as shown in (**A**). Genes displayed in grey boxes have no DEG data due to low or no expression in the corresponding tissues.

We further sought to determine if the DEGs arising from mtDNA supplementation could influence common functional pathways. The GO biological process terms that were commonly found comprised various metabolic processes and cell fate specification (Table S2). These GO terms were relatively high in the GO annotation hierarchy and often contained more than 20 child GO terms, part of the parent GO terms in a higher node, therefore providing a broader overview. Reactome pathway enrichment analysis provided more specific function commonly arising from mtDNA supplementation (Table 2). Glyoxylate metabolism and glycine degradation (R-HSA-389661) and interferon signalling (R-HSA-913531) were two major functions influenced in these three tissues (Table 2). For example, genes associated with these functional pathways tended towards downregulation in liver due to mtDNA supplementation (Figure 4A and Figure S3A). Genes in the cell cycle and DNA replication (R-HSA-68962, 69190 and 187577) and collagen biosynthesis pathways (R-HSA-1650814, 1474290 and 8948216) were predominantly upregulated in both heart and liver (Table 2) whilst genes in the cholesterol biosynthesis and vitamin metabolism pathways (R-HSA-191273, 196854 and 6806667) were predominantly downregulated in both brain and liver. The most affected function as a result of mtDNA supplementation varied amongst the tissues, for example, immune response and related functional pathways were most affected in brain, fatty acid, monosaccharide and acyl-CoA metabolic process in heart, and various tissue development pathways in liver (Figure 3). Overall, we identified the key functional pathways associated with mtDNA supplementation in adult tissues indicative of high mtDNA copy number.

### 2.4. The Effect of the Source of mtDNA for Supplementation on Gene Expression

Since we had generated heterologous and autologous mtDNA-supplemented-derived pigs (Figure 1), this provided us with an opportunity to investigate the effect of the source of mtDNA for supplementation, namely from third-party or sister oocytes, on transcriptome profiles. We identified a higher number of DEGs in brain (66 DEGs), liver (44) and heart (372) between the heterologous and autologous pigs (Tables S3–S5) than when controls were compared with both sets of mtDNA-supplemented pigs. One of the reasons could be gender bias in the pig groups. The heterologous group comprised one female and two male pigs, whilst the autologous group included only male pigs (Figure 1 and Table S1). It was evident from the PCA plots that female pigs have distinct transcriptome profiles (Figure 2C,D); however, we were not able to apply gender as a covariate factor in this analysis due to the lack of female pigs in the autologous group. This may have had a greater influence in the heart tissue analysis, as there was a higher degree of separation of the female samples from the male samples (Figure 2D). Amongst the DEGs in heart, 15 were linked to the X chromosome, whilst no X-chromosome-linked genes were found in control and overall mtDNA supplementation comparisons (Table 1 and Table S5), suggesting a gender-bias effect, at least in part. Brain and liver data seemed to be less affected, as we identified only one X-chromosome-linked DEG in liver.

In order to focus on the functional effects associated with the source of mtDNA for supplementation, we searched the common affected functional pathways amongst the three tissues. We found that GO biological process and Reactome terms commonly enriched in the DEGs of the three tissues were related to the TCA cycle and respiratory electron transport associated with mitochondrial function (Table 3, Tables S6 and S7). We also identified that non-integrin membrane-ECM interaction (R-HSA-3000171)-related genes were enriched in the DEGs of the three tissues. Genes associated with the TCA cycle pathway (R-HSA-71403) and respiratory electron transport pathway (R-HSA-611105) showed an upregulated trend in the brain and heart of the heterologous pigs (Figure 4B and Figure S3B), whilst we observed a trend towards downregulation in the autologous pigs. In liver, we observed the opposite trend, with downregulation in the heterologous pigs. These outcomes suggest that the source of mtDNA for supplementation has specific effects on the expression of genes associated with critical mitochondrial function in adult tissues.

### 2.5. Imprinted Genes Are Differentially Expressed in mtDNA-Supplemented-Derived Pig Heart and Liver

**Figure 5:**
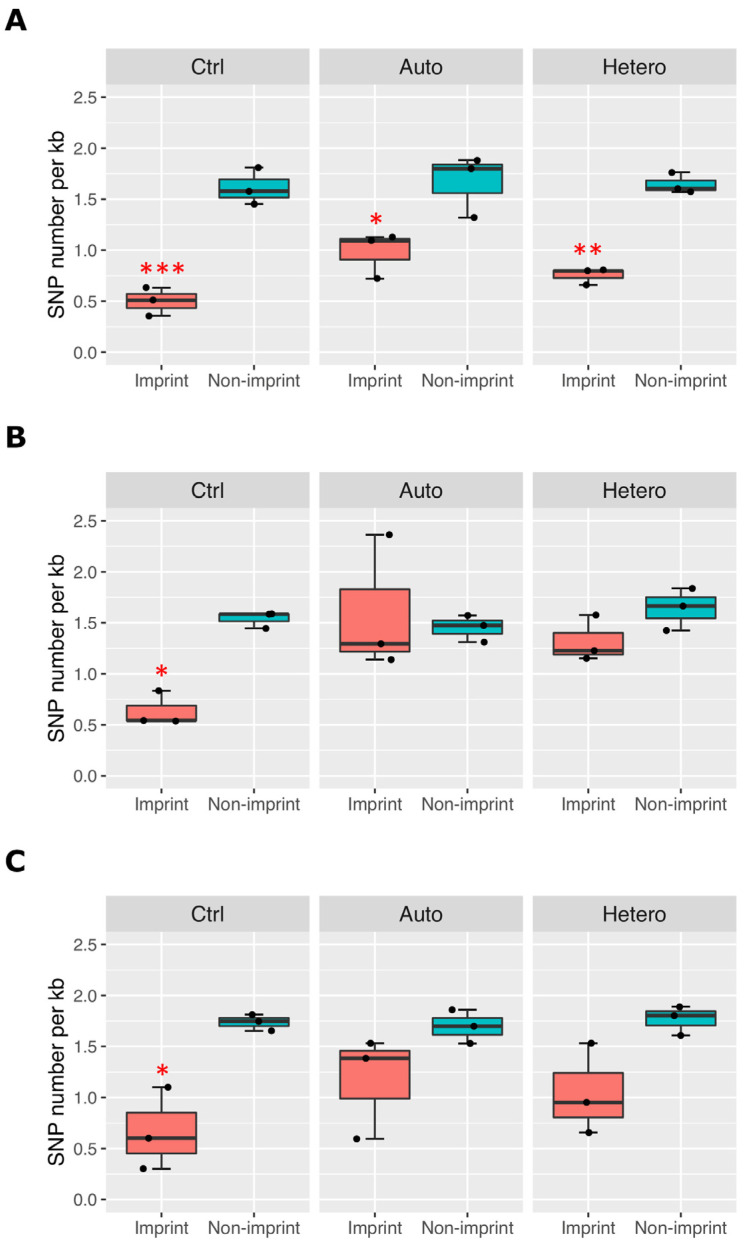
Biallelic expression of genes on imprinted loci in the tissues from control pigs (Ctrl) or pigs derived from autologous (Auto) and heterologous (Hetero) mtDNA supplementation. SNP frequency normalised by size of transcript (per kb) was measured as an indicator of biallelic expression for imprinted and non-imprinted genes from (**A**) brain, (**B**) heart and (**C**) liver RNAseq data. Significant differences identified by ANOVA and Tukey test are indicated by asterisks (*, *p* < 0.05; ** *p* < 0.01; ***, *p* < 0.001).

We have previously shown that allele-specific imprinted gene expression patterns were different between blastocysts generated with and without mtDNA supplementation [35]. We also demonstrated that single nucleotide polymorphism (SNP) frequency, calculated by SNP number divided by transcript length (kb), can be used as an indicator of mono-allelic or bi-allelic expression of genes at low and high frequency, respectively. For example, imprinted genes are biased to mono-allelic expression, resulting in lower SNP frequency, whilst non-imprinted genes are transcribed from both parental alleles, thus likely producing higher SNP frequency. This was evident in control pigs for all three tissues, showing significant differences between imprinted and non-imprinted genes (Figure 5). The difference was less significant in brain tissue from the mtDNA-supplemented-derived pigs (both autologous and heterologous), and the levels of SNP frequency between imprinted and non-imprinted genes in heart and liver were not significantly different. We also tested if there were any significant differences in SNP frequency between autologous and heterologous groups; however, no differences were found.

It is possible that low or high SNP frequency may simply reflect genetic variation of parents for specific genes and genomic regions. For example, for the imprinted gene *DIS3 like 3′-5′ exoribonuclease 2* (*DIS3L2*), we identified a total of 17 and 14 SNPs in the mtDNA-supplemented-derived pigs 3.4 and 12.4, respectively before bi-allelic SNP filtering (see Methods), whilst, in control pigs C7 and 26 we found only three SNPs. To minimise gene-specific distribution bias of the SNPs, we analysed a total transcript length of 52,177 bp for 14 imprinted genes and 735,642 bp for a further 210 non-imprinted genes which neighboured each imprinted gene that are included in Figure 5 (see Methods and Table S8). Taken together, we conclude that parental allele-specific imprinted gene expression patterns were disrupted in the tissues of the pigs derived from mtDNA supplementation through both autologous and heterologous means.

## 3. Discussion

In this study, we addressed the effects of mtDNA supplementation on gene expression in adult pig tissues that are known to contain high numbers of mtDNA copy. We found that mtDNA supplementation influenced glyoxylate metabolism and glycine degradation and interferon signalling pathways in brain, heart and liver tissues (Table 2). Interferon is a key component of the innate immune response, including inflammatory response, and the first line of defence against viral infection [36]. We have previously reported that mtDNA supplementation also affects the expression of genes associated with immune cell trafficking and inflammatory response pathways in blastocyst stage embryos [20,28]. Although we did not find changes in the expression of the same genes associated with interferon signalling pathways in the mtDNA-supplemented blastocysts, mtDNA supplementation of the oocyte at the time of fertilisation seems to affect immune responses in early development and maintains the effect in adult tissues.

Although we isolated purified populations of mitochondria for supplementation from immature oocytes following in vitro maturation (IVM) in order to generate mtDNA-supplemented-derived pigs [29], it might be possible that the mitochondria from these oocytes were damaged and/or stressed due to oocyte quality. Increased reactive oxygen species (ROS) and release of mtDNA from damaged mitochondria into the cytoplasm could activate various pro-inflammatory signalling pathways such as Toll-like receptor 9 or via cytosolic cGAS-STING [37,38]. Indeed, cytosolic oxidised mtDNA can also directly bind and activate the NLRP3 inflammasome [39]. Nevertheless, our previous reports have shown that these mitochondria generate similar levels of ATP through OXPHOS to the mitochondria from mature fertilisable oocytes [20] suggesting that mitochondrial integrity might not be an issue. Alternatively, inflammatory signals can be spread to neighbouring cells through mitochondrial transfer via nanotube tunnels, mtDNA transfer via extracellular vesicles and the cGAS-induced secondary messenger cGAMP [37]. These processes could explain the changes in expression in inflammatory response pathway genes in blastocysts [20,28]. Heteroplasmy (in this case the presence of two distinct mitochondrial genomes [22]) following heterologous third-party mtDNA supplementation could be one of the possible causes, especially given that the immune response may recognise this population of mtDNA as ‘foreign’ rather than ‘self’ mtDNA [40]. We have shown that supplemented heterologous mtDNA was detectable in tail samples from piglets and adult tissues (brain, calf muscle and heart), and levels of supplemented heterologous mtDNA varied amongst pigs and tissues but were at consistently low levels [29]. Heteroplasmy causes alterations in OXPHOS activity and mitochondrial ROS levels [22], which, in turn, modulate several signalling pathways, including RIG-I-like receptor (RLR) innate immune signalling [38,41]. Indeed, it has also been proposed that inherited mtDNA SNPs and somatic mutations could influence inflammatory pathways by imparting functional changes in mitochondria [38]. Furthermore, mitochondria also directly participate in RLR signalling, as mitochondrial antiviral signalling protein (MAVS) binds to RLRs and activates downstream nuclear factor kB and interferon regulatory factor signalling pathways for pro-inflammatory cytokine and type I interferon production [41]. Consequently, further investigation is required to assess immune response risks arising following oocyte cytoplasmic transfer, nuclear spindle and pronuclear transfer and mitochondrial supplementation.

Glyoxylate metabolism and the glycine degradation pathway were also commonly affected as a result of mtDNA supplementation in the three tissues tested (Table 2). In liver, for example, D-amino acid oxidase (DAO), which produces glyoxylate from glycine in peroxisomes, and glycine decarboxylase (GLDC), which degrades glycine as part of the glycine cleavage enzyme system in the mitochondrial matrix, were downregulated (Figure S3A), possibly resulting in accumulation of glycine. Glycine is an essential raw material for the synthesis of DNA and RNA and also major amino acids in mammals and is most abundant in collagen which represents 30% of the total protein in animals [42,43]. It can also improve immune responses; for example, glycine treatment prevents liver fibrosis by preventing the release of pro-inflammatory and pro-fibrogenic cytokines [44] and improves survival rate and liver function by controlling the production of pro-inflammatory or anti-inflammatory cytokines in the endotoxin-induced liver injury mouse model [45]. Therefore, changes in glyoxylate and glycine metabolism associated with mtDNA supplementation could influence a wide range of metabolism and physiological responses. Indeed, some of the responses associated with transcriptional changes were captured by biochemical and haematological assessments in mtDNA-supplemented-derived pigs [29]. For example, we have observed significant increases in cholesterol in pigs derived through both heterologous and autologous supplementation [29], and this was partly supported by the transcriptome analysis in this study as cholesterol biosynthesis in brain and liver was influenced by mtDNA supplementation (Table 2). In all, the addition of extra copies of mtDNA point to the reestablishment of interactions between the nuclear and the mitochondrial genomes where each genome seeks to establish a balance with the other –a process where co-operation between the two genomes would allow development to proceed and cells to function as best they can with likely alterations to anticipated developmental pathways that are evident in individual tissues [9].

Transcriptome comparisons between autologous and heterologous mtDNA-supplemented-derived pig tissues revealed that the TCA cycle and OXPHOS pathway genes were enriched in DEGs (Table 3, Figure 4 and Figure S3). Heteroplasmy influences metabolic processes and OXPHOS function, causing mitochondrial disease and inducing tumorigenesis when levels of heteroplasmy exceed a given threshold [46]. The levels of heteroplasmy in heterologous mtDNA-supplemented-derived pigs were not very high in the brain, tail and heart tissues that were tested, mostly lower than 2% at different variant call positions [29]. However, individual mtDNA mutations or haplotypes could accumulate to higher levels within individual cells [47] and possibly cause a biochemical defect, resulting in human diseases and disorders [48]. Our results suggest that even low levels of heteroplasmy associated with third-party mtDNA supplementation influence the transcription of nuclear encoded genes enriched for respiratory electron transport and TCA cycle activity likely as a result of early immune programming or disturbance shortly after the supplementation process (Table 3). Metabolites from the TCA cycle, such as acetyl-CoA and α-ketoglutarate, are co-factors that lead to the generation and modification of epigenetic marks, especially histone acetylation, histone methylation and DNA methylation [7,49]. Furthermore, different mtDNA haplotypes have been reported to show functional differences in terms of ATP generation through OXPHOS that influences cell metabolism and, at the same time, alter DNA methylation profiles and nuclear gene expression [50,51]. This is concerning if these changes in gene expression are associated with developmental and physiological defects, as several clinics worldwide have sought to introduce this procedure into clinical practice [23,24].

Our transgenerational study in mice derived from mtDNA-supplemented oocytes revealed a significant increase in litter size and the number of primordial follicles across three generations, and also showed a defect in cardiac structure in first- and second-generation offspring [52]. In our most recent study, anatomical, clinical, biochemical and haematological investigations of mtDNA-supplemented-derived pigs only exhibited minor differences and did not affect health and well-being of founders and progeny up to sexual maturity [29]. There were no differences in the DNA methylation profiles of imprint control regions for selected imprinted genes between control and mtDNA-supplemented-derived pigs [29]. In this study, we did not find any significant differences in expression levels for imprinted genes; however, we observed a significant difference in parental allele-specific imprinted gene expression patterns in mtDNA-supplemented-derived pigs, with shifts to biallelic expression (Figure 5). Assisted reproductive technologies, for example in vitro embryo culture, in vitro fertilisation (IVF) and intracytoplasmic sperm injection, affect imprinting gene methylation and expression in placenta and cord blood, compared to natural conception [30,33]. Embryos generated by IVF and cultured in medium showed biallelic expression of *H19*, whereas little paternal expression was observed in naturally conceived foetuses [31,53]. Since we did not find differences in imprinted gene expression pattern between autologous and heterologous mtDNA supplementation, this is not associated with heterologous transfer, but primarily as a result of adding extra copies of mtDNA which likely results from the changes to the genomic balance imposed by extra copies of mtDNA being introduced into the oocyte at the time of fertilisation. We have shown that mtDNA supplementation improved parental allele-specific imprinted gene expression patterns in blastocysts compared to non-supplemented blastocysts [35]; however, it may not be sufficient to recover levels similar to naturally conceived pigs.

Overall, we have demonstrated that mtDNA supplementation of oocytes has long-term effects by changing gene expression patterns in adult tissues. Immune response and glyoxylate metabolism pathways were commonly affected by mtDNA supplementation. The source of mtDNA for supplementation also influenced the expression of OXPHOS genes, suggesting a possible link between heterologous supplementation and OXPHOS activity. Consequently, although mtDNA-supplemented-derived pigs looked healthy and exhibited no developmental defects up to sexual maturity [29], further study is required to determine if gene expression patterns affect animal development and health especially during adulthood.

## 4. Materials and Methods

### 4.1. Tissue Collection from the Pigs

Tissues were collected from control (naturally mated pigs) and from pigs generated by supplementing oocytes at the time of fertilisation with extra copies of mtDNA packaged in purified populations of mitochondria from either sister (autologous) or third-party (heterologous) oocytes in conjunction with intracytoplasmic sperm injection (ICSI), as described [20,29]. Briefly, cumulus–oocyte complexes were collected from gilt ovaries and cultured in in vitro maturation medium. Matured metaphase II oocytes were collected and used for mtDNA-supplemented ICSI, as previously described [20,29]. Resultant zygotes were then transferred to post-pubertal Large White x Landrace gilts at 26 weeks of age as recipients for embryo transfer. Pregnant recipients were fed a commercial gestation diet and housed in pens. Farrowing of recipients was supervised and all piglets were processed as per standard colony protocol. Piglets and recipient gilts were housed in a temperature-controlled room, and piglets received a vaccination and were allowed access to a commercial standard diet as described [29]. Tissues were collected from identical locations from each animal at the time of autopsy, frozen in liquid N_2_ and stored in a −80 °C freezer until use.

### 4.2. RNA Extraction from Heart, Liver and Brain, RNAseq Library Construction and NGS

Total RNA was extracted from approximately 10 mg of *Sus scrofa* brain, heart and liver tissue using the RNeasy Mini Kit (QIAGEN, VIC, Australia), according to the manufacturer’s instructions. RNAseq library construction and next-generation sequencing were performed by the Australian Genome Research Facility (Melbourne, VIC, Australia). Briefly, tissue RNA samples from control pigs (n = 3), autologous mtDNA-supplemented-derived founder pigs (n = 3) and heterologous mtDNA-supplemented-derived founder pigs (n = 3) were used to generate RNAseq libraries. Depletion of rRNA in the RNAseq library was conducted using the Ribo-zero stranded protocol (Illumina Inc. San Diego, CA, USA). NGS libraries were sequenced using 150 bp paired-end sequencing chemistry TruSeq SBS Kit v3 reagents on an Illumina NovaSeq S1 platform.

### 4.3. RNAseq Data Analysis and Differentially Expressed Gene (DEG) Identification

Tissue RNAseq sample metadata can be found in Table S1. RNAseq raw fastq files were quality checked by ‘*fastqc*’ (version 0.11.9) [54], and trimming of adapters and quality filtering were then performed by ‘*fastp*’ (version 0.20.1) [55] with options: –*detect_adapter_for_pe*, *-q 20*, *–length_required 30*. Trimmed and quality-filtered paired-end reads were aligned to the *Sus scrofa* genome assembly Ensembl release 98 [56] using ‘*STAR*’ (version 2.7) [57] with default parameters. Gene expression was quantified by counting the number of reads aligned to each Ensembl gene model using ‘*featureCounts*’ (version 1.5.2) [58], and output results were assessed for mapping quality by MultiQC version 1.9 [59]. Summary statistics for the RNAseq data are shown in Table S1.

The Trimmed Mean of M-values (TMM) normalisation method from *edgeR* was applied to normalise read counts according to library size differences between samples. PCA was performed to visualise the summary of gene expression for all libraries and identify whether any factors associated with RNAseq library preparation contributed to variation in gene expression patterns. Genes with low expression were filtered out prior to DEG analysis, keeping genes with at least 1 count per million (CPM) reads in the three samples. Genes were considered differentially expressed if their FDR (false discovery rate) adjusted *p*-value was <0.1 (Tables S3–S5). DEGs were visualised by heatmap using the R packages ggplot2 [60] and *pheatmap* [61].

### 4.4. Functional Pathway Enrichment and Gene Network Analysis

Functional pathway enrichment analyses for DEGs were performed using the *GSEA* v4.2.3 [62] and Enrichment Map [63] as described [64]. Briefly, *Sus scrofa* Ensembl gene IDs were used to search for corresponding human orthologue gene symbols using the Ensembl BioMart database [65] and for pathway enrichment analysis input. The annotation gene set file, Human_GO_AllPathways_with_GO_iea_May_25_2022_symbol.gmt from Bader Lab gene sets collections (http://download.baderlab.org/EM_Genesets/current_release/ accessed on 16 April 2023), was used for *GSEA* analysis with a default FDR threshold of 0.25. The enrichment analysis results from GO biological process [34] and REACTOME pathway [66] were then visualised by the Enrichment Map app in Cytoscape [67]. For protein–protein interaction network analysis, the STRING [68] app within Cytoscape was used.

### 4.5. Bi-Allelic Expression Analysis of Genes in Imprinting Loci

Imprinted genes and neighbouring genes at the locus were analysed for the level of bi-allelic expression by SNP identification in the transcripts, as described [35]. *Sus scrofa* imprinted genes (https://www.geneimprint.com/site/genes-by-species.Sus+scrofa accessed on 16 April 2023) with sufficient read depth were selected for analysis. To this extent, we set a threshold of 15,000 reads or more per imprinted gene transcript from tissue RNAseq data sets. The 14 imprinted genes at 8 loci selected for investigation were: Chr1: *NDN* locus, Chr2: *KCNQ1* locus (including *DHCR7*), Chr6: *NOB1* locus, Chr9: *PEG10* locus (including *PPP1R9A*, *PON2* and *DLX5*), Chr11: *KBTBD6* locus, Chr14: *INPP5F* locus (including *TACC2*), Chr15: *DIS3L2* locus and Chr18: *COPG2* locus (including *MEST*). Total transcript length analysed for SNP identification was 52,177 bp for the 14 imprinted genes and 735,642 bp for 210 non-imprinted neighbouring genes.

SNPs were identified and counted by using ‘*samtools mpileup*’ for mapped reads pileup [69] and ‘*VarScan*’ version 2.3.8 [70] for variant sequence search. Imprinted gene locus sequences including 2 Mb upstream and downstream sequences of the *Sus scrofa* reference genome sequence Sscrofa11.1 accession No. GCF_000003025.6 were used for read pileup. Variant call was made by *VarScan* with the options: *--min-avg-qual* 20 *--min-var-freq* 0.01 *--min-reads2* 10, and the results were further filtered by keeping variant calls if there were >25 read counts per site. Furthermore, if there were more than three SNPs per transcript, they were kept for further analysis as confident SNPs. SNPs fewer than three per transcript were discarded as they might be associated with technical issues such as PCR and sequencing errors. SNPs with variant frequency between 20–80%, namely bi-allelic SNPs, were used for calculating SNP frequency, namely the number of SNP counts divided by transcript length (kb) (Table S8). An ANOVA test was carried out to see statistical significance associated with imprinting status and pig type. Sex of pig was included as a covariate factor. Results were presented by jittered-boxplot using the R packages *ggplot2* [60].

## Figures and Tables

**Table 1:** Differentially expressed genes between mtDNA-supplemented and control pig brain, heart and liver tissues.

Gene_Id ^a^	Symbol	Chromosome ^b^	Gene_Biotype	Description	Log2fc ^c^	Logcpm ^d^	*p* Value ^e^	FDR ^f^
***Brain***								
ENSSSCG00000002529		AEMK02000452.1	pseudogene		−2.6084	0.2896	2.52 × 10^−12^	3.88 × 10^−8^
ENSSSCG00000041050		2	lncRNA		−1.1361	5.0041	6.54 × 10^−10^	5.03 × 10^−6^
ENSSSCG00000008995	LRAT	8	protein_coding	lecithin retinol acyltransferase (Source: VGNC Symbol; Acc: VGNC: 89798)	−3.2222	0.9333	2.21 × 10^−9^	1.13 × 10^−5^
ENSSSCG00000000207		5	protein_coding		−1.8664	1.1183	7.49 × 10^−9^	2.88 × 10^−5^
ENSSSCG00000048719		4	lncRNA		−2.3907	2.7621	3.39 × 10^−8^	1.04 × 10^−4^
ENSSSCG00000037684	PDYN	17	protein_coding	prodynorphin (Source: VGNC Symbol; Acc: VGNC: 96477)	−1.8890	1.9398	1.20 × 10^−7^	3.08 × 10^−4^
ENSSSCG00000041461		AEMK02000489.1	lncRNA		−1.0474	6.2832	1.44 × 10^−6^	0.0032
ENSSSCG00000031141		9	protein_coding		0.7673	5.3265	4.02 × 10^−6^	0.0077
ENSSSCG00000014876	MYO7A	9	protein_coding	myosin VIIA (Source: VGNC Symbol; Acc: VGNC: 90533)	−1.2258	2.8836	1.04 × 10^−5^	0.0178
ENSSSCG00000010017	SMTN	14	protein_coding	smoothelin (Source: VGNC Symbol; Acc: VGNC: 93267)	−0.6804	5.0354	1.35 × 10^−5^	0.0207
ENSSSCG00000018076	tRNA-Ser	MT	Mt_tRNA	Product = tRNA-Ser	−1.1040	3.7806	1.66 × 10^−5^	0.0232
ENSSSCG00000018061	12S rRNA	MT	Mt_rRNA	product = 12S ribosomal RNA	−0.6911	7.5659	2.82 × 10^−5^	0.0362
ENSSSCG00000035731		1	lncRNA		−1.1817	3.2115	3.44 × 10^−5^	0.0407
ENSSSCG00000008997	FGB	8	protein_coding	fibrinogen beta chain (Source: VGNC Symbol; Acc: VGNC: 98927)	−1.3491	1.5436	3.96 × 10^−5^	0.0435
ENSSSCG00000043245		6	lncRNA		3.6467	0.8892	4.69 × 10^−5^	0.0481
ENSSSCG00000018071	tRNA-Ala	MT	Mt_tRNA	product = tRNA-Ala	9.4169	0.5293	5.40 × 10^−5^	0.0520
ENSSSCG00000024249	GABRR2	1	protein_coding	gamma-aminobutyric acid type A receptor subunit rho2 (Source: HGNC Symbol; Acc: HGNC: 4091)	3.9193	1.1744	6.64 × 10^−5^	0.0601
ENSSSCG00000008898		8	protein_coding	HOP homeobox (Source: VGNC Symbol; Acc: VGNC: 88932)	−0.6543	6.0757	8.68 × 10^−5^	0.0742
ENSSSCG00000044872		8	lncRNA		−2.0276	0.6183	9.28 × 10^−5^	0.0751
***Heart***								
ENSSSCG00000031876	TMEM159	3	protein_coding	transmembrane protein 159 (Source: HGNC Symbol; Acc: HGNC: 30136)	−1.6888	3.5787	9.09 × 10^−16^	1.25 × 10^−11^
ENSSSCG00000013513	PLIN5	2	protein_coding	perilipin 5 (Source: VGNC Symbol; Acc: VGNC: 91561)	−1.7601	3.7959	4.31 × 10^−11^	2.97 × 10^−7^
ENSSSCG00000002529		AEMK02000452.1	pseudogene		−2.5629	0.6598	4.22 × 10^−9^	1.94 × 10^−5^
ENSSSCG00000000207		5	protein_coding		−1.1382	3.2356	3.42 × 10^−6^	0.0118
ENSSSCG00000009498		11	protein_coding		3.8297	0.3891	8.24 × 10^−6^	0.0227
ENSSSCG00000018095	tRNA-Thr	MT	Mt_tRNA	product = tRNA-Thr	8.4889	−0.0175	1.38 × 10^−5^	0.0318
ENSSSCG00000045913		AEMK02000489.1	lncRNA		1.0243	4.0233	3.30 × 10^−5^	0.0648
ENSSSCG00000018070	tRNA-Trp	MT	Mt_tRNA	product = tRNA-Trp	9.7782	3.3464	4.20 × 10^−5^	0.0722
ENSSSCG00000007552		3	protein_coding		1.6328	3.3521	4.76 × 10^−5^	0.0729
ENSSSCG00000018071	tRNA-Ala	MT	Mt_tRNA	product = tRNA-Ala	9.4738	0.9580	5.62 × 10^−5^	0.0732
ENSSSCG00000014921	PRSS23	9	protein_coding	serine protease 23 (Source: VGNC Symbol; Acc: VGNC: 91880)	−0.6539	5.0841	5.85 × 10^−5^	0.0732
ENSSSCG00000003616	FAM167B	6	protein_coding	family with sequence similarity 167 member B (Source: VGNC Symbol; Acc: VGNC: 87925)	−0.7897	3.3686	7.56 × 10^−5^	0.0754
ENSSSCG00000010096	AIFM3	14	protein_coding	apoptosis inducing factor mitochondria associated 3 (Source: VGNC Symbol; Acc: VGNC: 85200)	−1.8782	2.3705	7.60 × 10^−5^	0.0754
ENSSSCG00000029268	TOPAZ1	13	protein_coding	testis and ovary specific PAZ domain containing 1 (Source: VGNC Symbol; Acc: VGNC: 94314)	1.0174	3.5161	7.67 × 10^−5^	0.0754
ENSSSCG00000050540		8	lncRNA		1.4389	2.6459	9.88 × 10^−5^	0.0874
ENSSSCG00000026686	PDZD9	3	protein_coding	PDZ domain containing 9 (Source: VGNC Symbol; Acc: VGNC: 91297)	1.0081	2.6808	1.04 × 10^−4^	0.0874
ENSSSCG00000018080	ATP8	MT	protein_coding	mitochondrially encoded ATP synthase membrane subunit 8 (Source: VGNC Symbol; Acc: VGNC: 99789)	4.4755	5.6610	1.08 × 10^−4^	0.0874
ENSSSCG00000024837	SYT12	2	protein_coding	synaptotagmin 12 (Source:VGNC Symbol; Acc: VGNC: 93681)	−1.1184	1.8219	1.16 × 10^−4^	0.0885
***Liver***								
ENSSSCG00000031474	PTK6	17	protein_coding	protein tyrosine kinase 6 (Source: VGNC Symbol; Acc: VGNC: 96517)	−5.5813	−0.0562	3.88 × 10^−17^	5.55 × 10^−13^
ENSSSCG00000003755	MCOLN2	6	protein_coding	mucolipin TRP cation channel 2 (Source: VGNC Symbol; Acc: VGNC: 90080)	3.4286	3.1445	5.10 × 10^−12^	3.65 × 10^−8^
ENSSSCG00000002529		AEMK02000452.1	pseudogene		−2.2307	1.4978	2.98 × 10^−10^	1.42 × 10^−6^
ENSSSCG00000000207		5	protein_coding		−1.3211	3.3023	1.82 × 10^−7^	6.41 × 10^−4^
ENSSSCG00000017220	OTOP3	12	protein_coding	otopetrin 3 (Source: VGNC Symbol; Acc: VGNC: 91095)	−3.2197	2.6204	2.24 × 10^−7^	6.41 × 10^−4^
ENSSSCG00000050540		8	lncRNA		1.7140	3.7928	4.50 × 10^−7^	0.0011
ENSSSCG00000016401	KIF1A	15	protein_coding	kinesin family member 1A (Source: HGNC Symbol; Acc: HGNC: 888)	3.6472	2.7749	6.73 × 10^−7^	0.0014
ENSSSCG00000049801		8	lncRNA		2.3897	1.9827	2.11 × 10^−6^	0.0038
ENSSSCG00000047296		8	lncRNA		2.5920	1.0668	2.41 × 10^−6^	0.0038
ENSSSCG00000034853		Y	protein_coding		4.2692	−0.5967	3.69 × 10^−6^	0.0053
ENSSSCG00000032896		10	protein_coding		2.4283	1.3265	8.02 × 10^−6^	0.0104
ENSSSCG00000040822	RERGL	5	protein_coding	RERG like (Source: VGNC Symbol; Acc: VGNC: 92218)	2.7953	−0.0188	1.73 × 10^−5^	0.0206
ENSSSCG00000001484	TINAG	7	protein_coding	tubulointerstitial nephritis antigen (Source: VGNC Symbol; Acc: VGNC: 93998)	−2.2315	0.6368	3.09 × 10^−5^	0.0340
ENSSSCG00000018070	tRNA-Trp	MT	Mt_tRNA	product = tRNA-Trp	9.4671	0.7267	5.85 × 10^−5^	0.0598
ENSSSCG00000046246		7	pseudogene		2.5585	0.3769	9.18 × 10^−5^	0.0876
ENSSSCG00000015002	ELMOD1	9	protein_coding	ELMO domain containing 1 (Source: VGNC Symbol; Acc: VGNC: 87652)	5.3844	−0.2513	1.03 × 10^−4^	0.0912
ENSSSCG00000004291	NT5E	1	protein_coding	5′-nucleotidase ecto (Source: VGNC Symbol; Acc: VGNC: 90925)	1.2377	2.9849	1.10 × 10^−4^	0.0912
ENSSSCG00000003603	COL16A1	6	protein_coding	collagen type XVI alpha 1 chain (Source: VGNC Symbol; Acc: VGNC: 86867)	1.4794	3.3102	1.15 × 10^−4^	0.0912
ENSSSCG00000035297	ISG12(A)	7	protein_coding	putative ISG12(a) protein (Source: NCBI gene (formerly Entrezgene); Acc: 100153902)	−1.5001	4.6091	1.24 × 10^−4^	0.0937

^a^, Ensembl (https://m.ensembl.org/index.html accessed on 16 April 2023) gene ID. ^b^, Chromosome or scaffold number where gene is located. ^c^, Log2-fold change relative to ICSI blastocyst. ^d^, Mean value of log2 count per million reads. ^e^, Raw *p*-value. ^f^, False discovery rate.

**Table 2:** Reactome terms commonly enriched in the comparison between control and mtDNA-supplemented pig tissue transcriptome data for more than two tissues. False discovery rate (FDR) *p*-values for enrichment analysis are shown.

Reactome_ID	Reactome_Term	Brain	Heart	Liver
389661	GLYOXYLATE METABOLISM AND GLYCINE DEGRADATION	0.2416	0.0779	0.2376
909733	INTERFERON ALPHA BETA SIGNALLING	0.1460	0.0281	0.2452
913531	INTERFERON SIGNALLING	0.2492	0.0783	0.1927
877300	INTERFERON GAMMA SIGNALLING	0.0625	0.1082	na
68962	ACTIVATION OF THE PRE-REPLICATIVE COMPLEX	na	0.2440	0.0713
69190	DNA STRAND ELONGATION	na	0.1688	0.2299
114508	EFFECTS OF PIP2 HYDROLYSIS	na	0.2386	0.2465
187577	SCF(SKP2)-MEDIATED DEGRADATION OF P27 P21	na	0.1373	0.0031
191273	CHOLESTEROL BIOSYNTHESIS	0.2346	na	0.0004
196854	METABOLISM OF VITAMINS AND COFACTORS	0.1906	na	0.2291
1474290	COLLAGEN FORMATION	na	0.2497	0.0000
1650814	COLLAGEN BIOSYNTHESIS AND MODIFYING ENZYMES	na	0.2481	0.0000
5619115	DISORDERS OF TRANSMEMBRANE TRANSPORTERS	na	0.2397	0.2397
6806667	METABOLISM OF FAT-SOLUBLE VITAMINS	0.0052	na	0.1915
8940973	RUNX2 REGULATES OSTEOBLAST DIFFERENTIATION	0.2115	na	0.1644
8941326	RUNX2 REGULATES BONE DEVELOPMENT	0.1433	na	0.2184
9658195	LEISHMANIA INFECTION	0.2358	na	0.2205
9662851	ANTI-INFLAMMATORY RESPONSE FAVOURING LEISHMANIA PARASITE INFECTION	0.1319	na	0.1506
9664323	FCGR3A-MEDIATED IL10 SYNTHESIS	0.2429	na	0.0510
9664433	LEISHMANIA PARASITE GROWTH AND SURVIVAL	0.1316	na	0.1489
R-HSA-2173782	BINDING AND UPTAKE OF LIGANDS BY SCAVENGER RECEPTORS	na	0.1789	0.1650
R-HSA-8948216	COLLAGEN CHAIN TRIMERISATION	na	0.1823	0.0000
R-HSA-983189	KINESINS	na	0.2124	0.2295

na, not available.

**Table 3:** Reactome terms commonly enriched in the comparison between heterologous and autologous mtDNA-supplemented-derived pig tissue transcriptome for more than two tissues, analysed by gene sequence enrichment analysis (GSEA). FDR *p*-values for enrichment analysis are shown. Top 30 terms are listed from Table S7.

Reactome_ID	Reactome_Term	Brain	Heart	Liver
R-HSA-1428517.1	THE CITRIC ACID (TCA) CYCLE AND RESPIRATORY ELECTRON TRANSPORT	0.0083	0.0198	0.0781
163200	RESPIRATORY ELECTRON TRANSPORT, ATP SYNTHESIS BY CHEMIOSMOTIC COUPLING, AND HEAT PRODUCTION BY UNCOUPLING PROTEINS	0.0653	0.0139	0.0673
R-HSA-611105.3	RESPIRATORY ELECTRON TRANSPORT	0.0255	0.0365	0.1497
R-HSA-3000171.3	NON-INTEGRIN MEMBRANE-ECM INTERACTIONS	0.1462	0.0184	0.1450
5173214	O-GLYCOSYLATION OF TSR DOMAIN-CONTAINING PROTEINS	na	0.0000	0.0579
9031628	NGF-STIMULATED TRANSCRIPTION	na	0.0529	0.0057
5083635	DEFECTIVE B3GALTL CAUSES PPS	na	0.0000	0.0592
R-HSA-1296071.2	POTASSIUM CHANNELS	na	0.0497	0.0123
198725	NUCLEAR EVENTS (KINASE AND TRANSCRIPTION FACTOR ACTIVATION)	na	0.0631	0.0053
R-HSA-445355.6	SMOOTH MUSCLE CONTRACTION	na	0.0104	0.0597
R-HSA-5389840.1	MITOCHONDRIAL TRANSLATION ELONGATION	na	0.0154	0.0707
5419276	MITOCHONDRIAL TRANSLATION TERMINATION	na	0.0077	0.0946
R-HSA-3906995.3	DISEASES ASSOCIATED WITH O-GLYCOSYLATION OF PROTEINS	na	0.0010	0.1056
418597	G ALPHA (Z) SIGNALLING EVENTS	na	0.0584	0.0598
R-HSA-77289.5	MITOCHONDRIAL FATTY ACID BETA-OXIDATION	0.0539	0.0656	na
R-HSA-187037.2	SIGNALLING BY NTRK1 (TRKA)	na	0.0871	0.0418
R-HSA-1474228.4	DEGRADATION OF THE EXTRACELLULAR MATRIX	na	0.0132	0.1168
69017	CDK-MEDIATED PHOSPHORYLATION AND REMOVAL OF CDC6	na	0.1258	0.0089
R-HSA-69610.4	P53-INDEPENDENT DNA DAMAGE RESPONSE	na	0.1223	0.0214
R-HSA-156842.2	EUKARYOTIC TRANSLATION ELONGATION	na	0.0000	0.1449
R-HSA-5368287.1	MITOCHONDRIAL TRANSLATION	na	0.0049	0.1403
R-HSA-156902.2	PEPTIDE CHAIN ELONGATION	na	0.0000	0.1475
R-HSA-180585.1	VIF-MEDIATED DEGRADATION OF APOBEC3G	na	0.1197	0.0304
R-HSA-69601.3	UBIQUITIN-MEDIATED DEGRADATION OF PHOSPHORYLATED CDC25A	na	0.1197	0.0324
R-HSA-977444.3	GABA B RECEPTOR ACTIVATION	na	0.1299	0.0255
R-HSA-450408.3	AUF1 (HNRNP D0) BINDS AND DESTABILISES MRNA	na	0.1455	0.0106
R-HSA-5368286.1	MITOCHONDRIAL TRANSLATION INITIATION	na	0.0048	0.1525
R-HSA-69613.2	P53-INDEPENDENT G1 S DNA DAMAGE CHECKPOINT	na	0.1405	0.0182
5619084	ABC TRANSPORTER DISORDERS	na	0.1279	0.0324
R-HSA-6799198.1	COMPLEX I BIOGENESIS	na	0.0592	0.1032

na, not available.

## Data Availability

The datasets supporting the conclusions of this article are available in the NCBI Sequence Read Archive (https://www.ncbi.nlm.nih.gov/sra accessed on 16 April 2023) under the BioProject ID PRJNA823749.

## Supplementary Materials

The following supporting information can be downloaded at: https://www.mdpi.com/article/10.3390/ijms24087545/s1.
