# Supplementary material for: Mitochondrial DNA Supplementation of Oocytes Has Downstream Effects on the Transcriptional Profiles of Sus scrofa Adult Tissues with High mtDNA Copy Number
Source: Int J Mol Sci. 2023 Apr 19;24(8):7545. doi: 10.3390/ijms24087545 (PMC10140937; doi:10.3390/ijms24087545)
Supplement: Supplementary file 1 [file ijms-24-07545-s001.zip › ijms-2256042-supplementary/ijms-2256042 Sup Figures.pdf]

Supplementary Figures

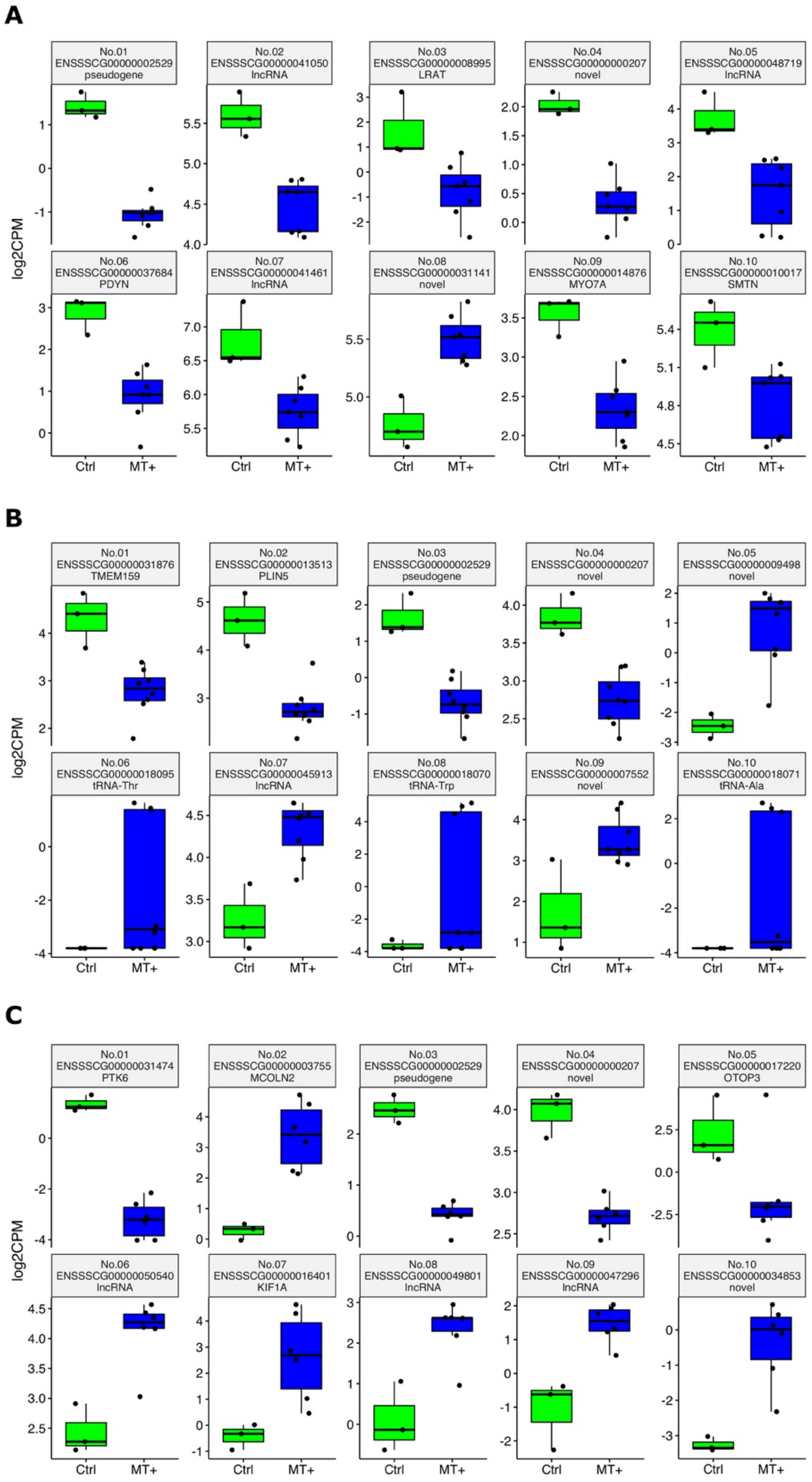

Figure S1. Top 10 DEGs between control and mtDNA supplemented-derived pig tissues, (**A**) brain, (**B**) heart and (**C**) liver. Levels of expression were plotted on the *y*-axis as log2CPM value and presented by jittered-boxplot. The full DEG list is shown in Table 1.

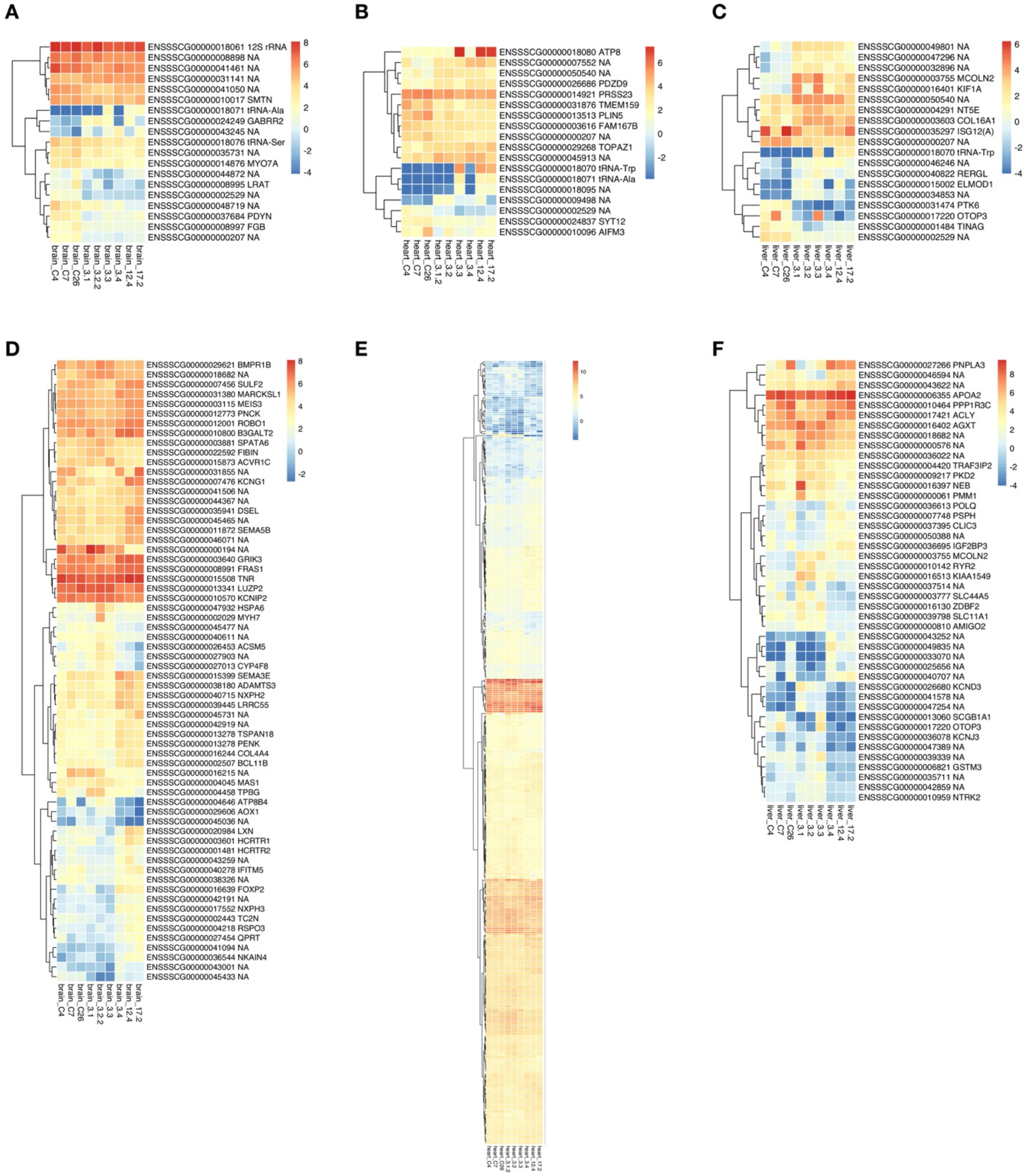

Figure S2. Expression of DEGs between control and mtDNA supplemented-derived pig (**A**) brain; (**B**) heart and (**C**) liver tissues and DEGs between heterologous and autologous mtDNA supplemented-derived pig (**D**) brain; (**E**) heart and (**F**) liver tissues. Expression of DEGs was displayed by heatmap. Rows show individual genes ordered by hierarchical clustering based on gene expression patterns. Each tile in the main matrix represents the levels of expression of a single gene in a single RNAseq data set. Colour of tile indicates levels of expression in TMM-normalized logCPM, and a scale is presented on the right. A list of DEGs is found in Tables 1 and S3 - S5. NA indicates no annotation was available for this gene region.

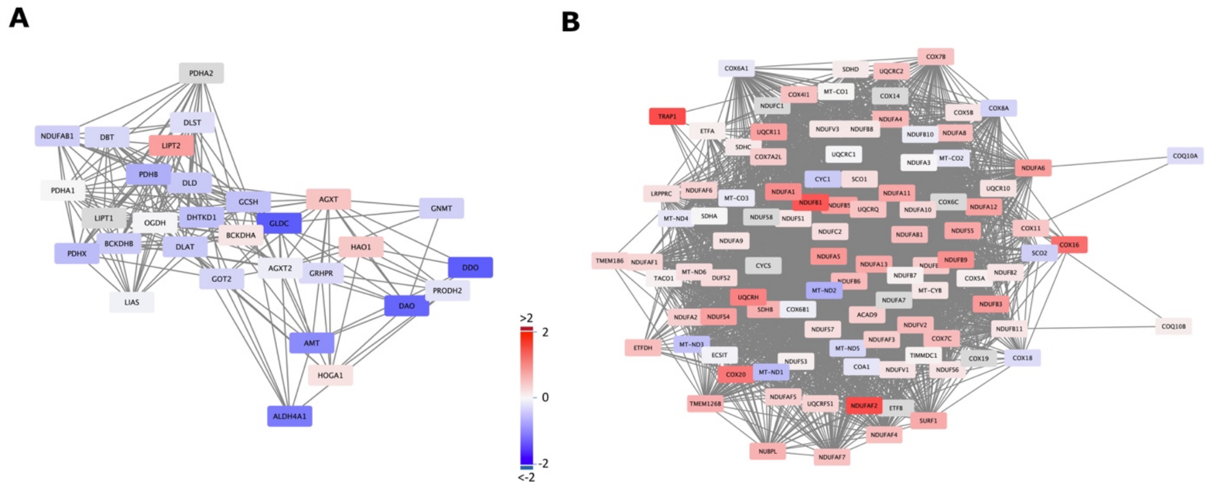

Figure S3. Gene network and differential gene expression of functional pathways identified by enrichment analysis. (**A**) The glyoxylate metabolism and glycine degradation pathway (R-HSA-389661) genes expressed in tissues were used to construct a protein interaction network by STRING. The levels of differential gene expression between control pig and mtDNA supplemented-derived liver tissues are shown as fold change relative to control by colour scale (right). (**B**) The respiratory electron transport pathway (R-HSA-611105) genes expressed in tissues were used to a construct protein interaction network. The levels of differential gene expression between heterologous and autologous mtDNA supplemented-derived pig heart are shown as fold change relative to autologous mtDNA supplementation by colour scale. The same colour scale was used as shown in (**A**). Genes in grey boxes have no DEG data due to low or no expression in their corresponding tissues.
